# Maintaining HIV testing and treatment services in Zambia during COVID-19: a story of success and resilience

**DOI:** 10.1080/16549716.2023.2175992

**Published:** 2023-02-21

**Authors:** Tina Chisenga, Menard Chihana, Paul Chishimba, Lastone Chitembo, Lloyd Mulenga, Andrew Silumesii, David Maman, Cheryl Johnson

**Affiliations:** a Ministry of Health Zambia, Department of Communicable Diseases, Lusaka, Zambia; b World Health Organization, Global HIV, Hepatitis and STI Programmes, Geneva, Switzerland; c Ministry of Health Zambia, Department of Monitoring and Evaluation, Lusaka, Zambia; d World Health Organization, Department of HIV, Viral Hepatitis and STIs, Lusaka, Zambia; e Department of Infectious Disease, University Teaching Hospital, Lusaka, Zambia; f The Global Fund, Technical Advice and Partnership Department, Geneva, Switzerland

**Keywords:** Covid-19, HIV, ART, health service delivery, HIV testing services

## Abstract

**Background:**

Coronavirus disease 2019 (COVID-19) is caused by a virus called severe acute respiratory syndrome coronavirus. As countries struggled to control the spread of the virus through among other measures closure of health facilities, repurposing of health care workers, and restrictions on people’s movement, HIV service delivery was affected.

**Objectives:**

To assess the impact of COVID-19 on HIV service delivery in Zambia by comparing uptake of HIV services before and during COVID-19.

**Methods:**

We used repeated cross-sectional quarterly and monthly data on HIV testing, HIV positivity rate, people living with HIV initiating ART and use of essential hospital services from July 2018 to December 2020. We assessed quarterly trends and measured proportionate changes comparing periods before and during COVID-19 divided into three different comparison time frames: (1) annual comparison 2019 versus 2020; (2) April to December 2019 versus same period in 2020; and (3) Quarter 1 of 2020 as base period versus each of the other quarters of year 2020.

**Results:**

Annual HIV testing dropped by 43.7% (95%CI 43.6–43.7) in 2020 compared to 2019 and was similar by sex. Overall, annual recorded number of newly diagnosed PLHIV fell by 26.5% (95% CI 26.37–26.73) in 2020 compared to 2019, but HIV positivity rate was higher in 2020, 6.44% (95%CI 6.41–6.47) compared to 4.94% (95% CI 4.92–4.96) in 2019. Annual ART initiation dropped by 19.9% (95%CI 19.7–20.0) in 2020 compared to 2019 while use of essential hospital services dropped during the early months of COVID-19 April to August 2020 but picked up later in the year.

**Conclusion:**

While COVID-19 had a negative impact on health service delivery, its impact on HIV service delivery was not huge. HIV policies that were implemented before COVID-19 on testing made it easier to adopt COVID-19 control measures and to continue providing HIV testing services without much disruption.

## Introduction

Coronavirus disease 2019 (COVID-19) is a respiratory infectious disease caused by a virus called severe acute respiratory syndrome coronavirus 2 (SARS-CoV-2). The virus was first detected in Wuhan City, Hubei Province, China [1], and quickly spread throughout the world infecting hundreds of millions of people. As of mid-2022, over 550 million people had been infected and over 6.3 million had died from the virus, with the United States of America being the most affected country in terms of both number of people infected and fatalities [2]. In Africa, particularly sub-Saharan Africa, Zambia was the 4^th^ worst affected country in both number of infections and fatalities after South Africa, Ethiopia and Kenya [2]. The World Health Organisation (WHO) declared COVID-19 a Public Health Emergency of International Concern on 30 January 2020 [1,3] and later a pandemic on 11 March 2020 [1,4].

The spread of SARS-CoV-2 caused massive disruptions in many countries across the world as countries struggled to contain its spread. In many countries, the pandemic affected health service delivery, particularly HIV services [5–8]. In Zambia, patient zero diagnosed with SARS-CoV-2 was announced on 18 March 2020 [9,10]. By mid-2022, Zambia had recorded over 325 000 infections, 4 000 fatalities [2] and experienced two complete waves of the virus spread. The first wave came between July and September 2020, the second wave between December 2020 and April 2021, and by the time data for this article was being prepared, the country was experiencing a third wave which started in June 2021 [11]. Like in many other African countries, COVID-19 in Zambia did not spread to levels experienced in Europe and America. Nonetheless, to minimise the risk of virus spread, the government implemented a series of preventive measures. Among others, the measures included: restriction of non-essential travel outside district boundaries, mandatory wearing of personal protective equipment in public places i.e. face masks, encouraging institutions to ensure no crowding at workstations and service points, maintaining good hand hygiene practices at workplaces and in public places, enforcing carrying capacity in public transport and encouraging people to observe social distancing [12–14]. These measures coupled with people’s fears of catching the virus meant that many people were forced to stay at home.

COVID-19 could not have come at a critical time in Zambia. In 2019, HIV prevalence was estimated at 6.9% [15], and in 2020 the country achieved all the steps of UNAIDS 90-90-90 targets [16] which stated that by 2020, 90% of people living with HIV (PLHIV) should know the status, 90% of those aware of their status should be on antiretroviral therapy (ART) and 90% of those on ART should be virally suppressed [17]. While focusing on achieving the UNAIDS targets [17], the country implemented several policies to improve testing coverage through expansion of index client (a person living with HIV) testing which meant testing the contacts (e.g. sexual partners, children under 15 years old) of an index client [18,19], provider-initiated testing and counselling (PITC), voluntary counselling and testing (VCT), mobile testing to target key populations with a focus on retesting the negatives and piloting on HIV self-testing [20]. In addition, the country increased provision of HIV testing at sites where people came for voluntary medical male circumcision (VMMC), sexually transmitted infection (STI) testing and blood testing [21]. Men were specifically targeted to ensure they caught up with females in terms of HIV status awareness and ART initiation. Testing programmes at workplaces, at men’s Insaka social gatherings where men meet to discuss social issues and encouraging women to bring their partners to antenatal clinic (ANC) to access testing services [20,21] were some of the strategies identified to reach out to more males. To ensure that those who tested positive were initiated on ART, the country implemented the WHO universal treatment guidelines [22] for all PLHIV to be initiated on ART regardless of CD4 cell count.

When evidence from studies on HIV testing indicated that more than 50% of new HIV tests already tested positive before [23], it strengthened the idea to rethink and focus on how HIV testing is conducted. Zambia Ministry of Health (MOH), together with its implementing partners, the United States Agency for International Development (USAID) and The President’s Emergency Plan for AIDS Relief (PEPFAR), started concentrating on reducing over testing and directing their efforts to targeted testing. Through targeted testing, index testing was being promoted. They developed HIV risk screening tool to target those at higher risk of contracting HIV with an aim of increasing positivity yield (proportion of positives per number of tests).

There are several pathways through which COVID-19 can affect HIV testing and care services. Similar to disruptions caused by Ebola outbreak, and those experienced during COVID-19, the pandemic affected HIV service delivery through closure of health facilities [24,25], repurposing of health care workers [26], shifting of general hospital resources towards COVID-19 response, people’s fears to contract the virus [27], government-imposed restrictions on movement of people and public transport disruptions [28–30]. All these resulted in reduced visits to the hospital to seek outpatient department (OPD) services.

With many COVID-19 preventive protocols requiring minimal contact between people, decongesting of health facilities and strict observance of hygiene practices, Zambia had already started preparing for such through expansion of self-testing, taking testing outside health facilities through focus on targeted and index testing and stopping community door-to-door testing [15,20]. These were being implemented not knowing what COVID-19 would bring which means that they were already a step ahead in preparing for the implications of COVID-19 in HIV testing and care services. We aimed to show the resilience of the country’s health service delivery in dealing with COVID-19 by assessing its impact on HIV testing and care services. We describe and compare the uptake of these services before and during COVID-19.

## Methods

### Study setting

Zambia is a landlocked lower-middle-income country in the Southern African region bordered by 8 countries (Angola, Botswana, Democratic Republic of Congo, Malawi, Mozambique, Namibia, Tanzania, and Zimbabwe). Its 2020 population was estimated at 18.4 million [31]. The country is divided into 10 provinces with Lusaka and Copperbelt provinces harbouring most of the population. Over 50% of the nation’s population live below the poverty line with many poor rural dwellers relying on subsistence farming as a source of food and income. Zambia has a generalised HIV epidemic, and in 2020 about 1.5 million individuals were estimated to be living with HIV [32]. In 2020 the country achieved the UNAIDS 90-90-90 targets [16] and is currently focusing on achieving the UNAIDS 95-95-95 targets to end HIV as a public health problem by the year 2030.

### Data sources

We used routinely collected quarterly aggregated data from District Health Information System (DHIS). HIV testing, ART and other health indicators data from all service providers are captured and recorded in DHIS together with important demographics data such as age, sex and area of residence.

### Statistical methods

Repeat cross-sectional study design was used to examine the impact of COVID-19 on HIV service delivery. We examined the following indicators: HIV testing numbers, HIV positivity rates, number of PLHIV initiating on ART and number of patients who were using essential hospital services, i.e. hospital admissions and OPD services.

We used quarterly data, first quarter; January to March, second quarter; April to June, third quarter; July to September, and fourth quarter; October to December on HIV testing and ART services. Analysis period was from July 2018 to December 2020. To assess the impact of COVID-19 on uptake or access to HIV testing and care services, two time periods (pre- and during COVID-19) were defined into three different comparison time frames as follows: (1) Annual comparison between 2019 (year without COVID-19) and 2020 (year during COVID-19). (2) April 2019 to December 2019 versus same period in 2020 (to compare period without COVID-19 in 2019 with similar period during COVID-19 in 2020). (3) Q1 of 2020 as base period without COVID-19 versus each of the other quarters (Q2-Q4) as periods with COVID-19. We used monthly data for the year 2020 for OPD and hospital admissions to measure the impact on use of essential hospital services.

We assessed quarterly HIV testing trends at national and provincial level stratified by sex and age group. Age group was divided into two categories, ‘children 1–14 years and adults 15+ years old’, while antenatal data for pregnant women were analysed as a separate category. We assessed provincial HIV testing and positivity trends to understand how different provinces were affected. We also examined trends in HIV positivity rate among those who were tested. We further looked at trends in number of people initiating on ART.

In addition, we measured the proportionate changes in HIV testing and PLHIV initiating on ART comparing the two periods according to each of the three defined periods above. Finally, we assessed the impact of COVID-19 on ANC visits and use of essential hospital services at OPD. Significance of change in proportions between the comparison periods were assessed using the 95% Confidence Interval (CI) and analysis was done using Microsoft Excel and STATA 16 (Stata Corp, Lakeway Drive College Station, Texas, USA).

The study obtained ethics approval from the Excellence in Research Ethics and Science (ERES) Converge Ethical review Board in Zambia. Consent from the individuals was not required as this was routinely collected aggregate data.

## Results

There were about 10.3 million records on testing between Q3 of 2018 and Q4 of 2019. Of these, 4.5 million (43.7%) were from 2019, and 2. 5 million (24.3%) were from 2020. About 1.5 million records (14.6%) on testing were for under 15 years, and 3.6 million records were for adult males 15+ years old. Total number of records who tested HIV positive was 509 693, of which 201 557 (39.5%) were males and 223 385 records (43.8%) were from 2019, while 164 079 (32.2%) were from 2020. Total number who initiated ART were 530 095, of which 202 496 (38.2%) were adult males and 225 095 (42.5%) were from 2019, while 180 398 (34.0%) were from 2020.

**Figure 1.**
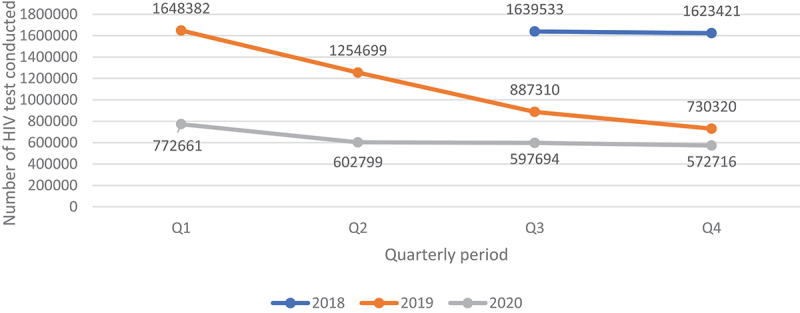
Quarterly HIV testing trends between July 2018 and December 2020.

**Figure 2.**
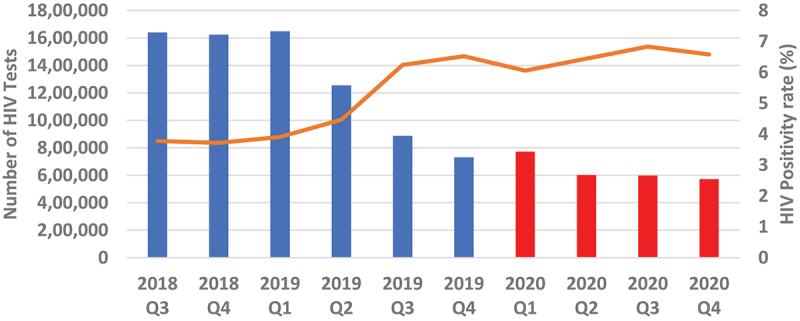
Showing overall quarterly HIV testing numbers and positivity rate from July 2018 to December 2020.

**Figure 3.**
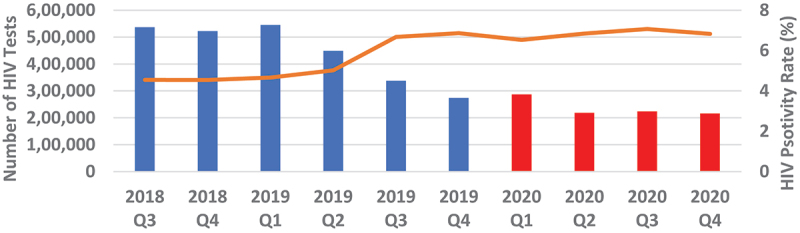
Quarterly HIV testing numbers and HIV positivity rate for males 15+ years from July 2018 to December 2020.

**Figure 4.**
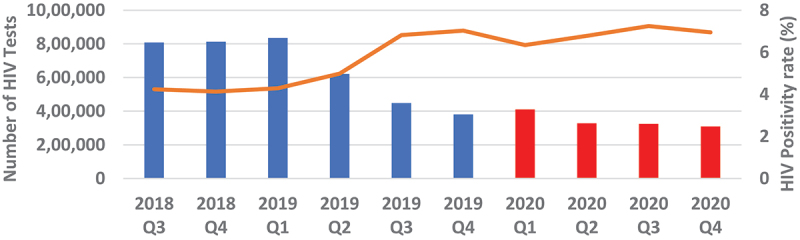
Quarterly HIV testing numbers and HIV positivity rate for females 15+ years from July 2018 to December 2020.

**Figure 5.**
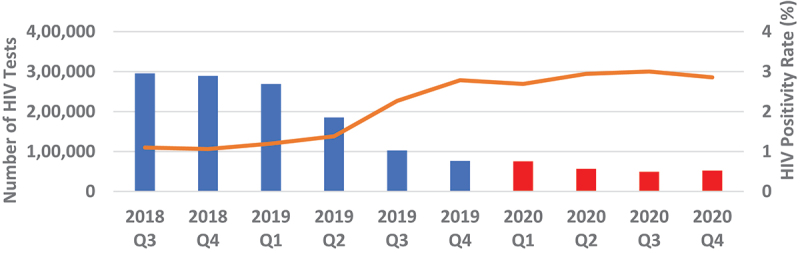
Quarterly HIV testing numbers and HIV positivity rate for under 15 years from July 2018 to December 2020.

**Figure 6.**
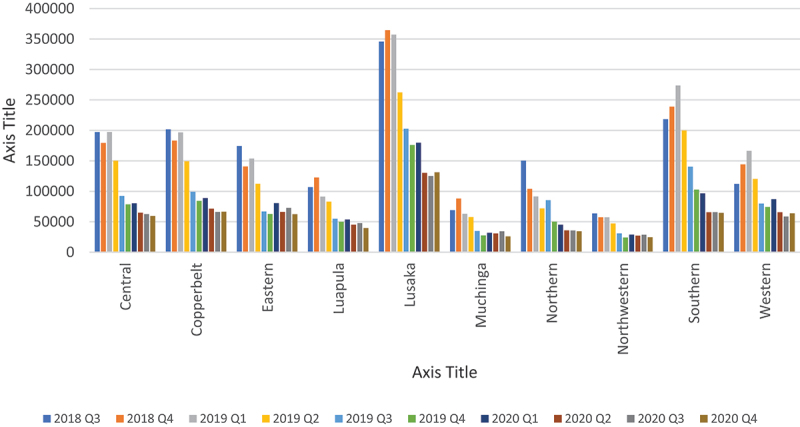
Showing quarterly HIV testing trends by province from Q3 of 2018 to Q4 2020.

Overall, quarterly HIV testing numbers were high reaching slightly above 1.6 million from Q3 of 2018 to Q1 of 2019 followed by a sudden drop in testing by 23.9% to around 1.25 million in Q2 of 2019 which continued to decline to around 730 000 in Q4 of 2019, representing a further 41.8% drop from Q2 of 2019. Testing numbers increased slightly in Q1 of 2020 before dropping again in Q2, where it stabilised thereafter to around 600 000 (Figures 1 and 2). Testing trends for adult males and females mirrored the overall trend (Figures 3 and 4). For children under 15, however, testing numbers were falling slowly up to Q1 of 2019 before unexpectedly taking a steep fall of about 61.9% between Q1 and Q3 of 2019 and continued with small declines to Q1 of 2020, then stabilising afterwards (Figure 5). HIV testing trends at provincial level showed that in all provinces the decline in testing numbers started before COVID-19 hit the country. While in provinces such as Eastern, Luapula, Muchinga and North-western their last substantial drop in testing was in Q3 of 2019, the other provinces continued to record substantial declines until Q2 of 2020 (Figure 6).

Annual HIV testing dropped by about 43.7% (95% Confidence Interval (43.6–43.7)) comparing 2019 with 2020. Comparing Q2-Q4 2020 period during COVID-19 to same period pre-COVID-19, we saw a drop of 38.3% (95% CI 38.2–38.3) in testing. Comparing Q1 of 2020 as a base pre-COVID-19 period with each of the other quarters in 2020, there was a drop of 22.0% (95% CI 21.9–22.1) in testing in Q2 and 25.9% (95% CI 25.8–26.0) in Q4 compared to Q1. Adult males recorded annual drop in testing numbers of 41.1% (95% CI 41.0–41.2) and adult females of 40.0% (95% CI 39.9–40.0) in 2020 when compared to testing in 2019 and a drop of 37.9% (95% CI 37.8–38.0) for adult males and 33.7% (95% CI 33.6–33.7) for adult females in Q2-Q4 of 2020 when compared to the same period in 2019. The biggest drop in testing, however, was reported among children under 15 years old with a 63.1% (95% CI 62.9–63.2) drop in annual testing in 2020 when compared to 2019 and 56.6% (95% CI 56.4–56.7) drop in Q2-Q4 of 2020 compared to same period in 2019. Pregnant women attending ANC were the least affected, with an 8.7% (95% CI 8.6–8.7) drop in annual testing in 2020 compared to 2019 (Table 1).

**Table 1.** Showing annual and quarterly proportion changes in testing by sex and age group.

Variable name/Period	Overall HIV testing	% change (95% CI)	Testing among Men 15+	% change (95% CI)	Testing among non-pregnant female 15 +	% change (95% CI)	Testing among pregnant female	% change (95% CI)	Testing among under 15	% change (95% CI)
2019	4520711		1604070		2283765		634246		632876	
2020	2545870	43.7 (43.6–43.7)	945215	41.1 (41.0–41.2)	1370951	40.0 (39.9–40.0)	579313	8.7 (8.6–8.7)	233735	63.1 (62.9–63.2)
April–December 2019	2872329		1059459		1448673		460137		364179	
April–December 2020	1773209	38.3 (38.2–38.3)	658110	37.9 (37.8–38.0)	960897	33.7 (33.6–33.7)	428110	7.0 (6.0–7.0)	158233	56.6 (56.4–56.7)
2020 Q1	772661		287105		410054		151203		75502	
2020 Q2	602799	22.0 (21.9–22.1)	218480	23.9 (23.7–24.1)	327626	20.1 (20.0–20.2)	143085	5.4 (5.3–5.5)	56693	24.9 (24.6–25.2)
2020 Q3	597694	22.6 (22.6–22.7)	223757	22.1 (21.9–22.2)	324594	20.8 (20.7–21.0)	143702	5.0 (4.9–5.1)	49343	34.6 (34.3–35.0)
2020 Q4	572716	25.9 (25.8–26.0)	215873	24.8 (24.7–25.0)	308677	24.7 (24.6–24.9)	141323	6.5 (6.4–6.7)	52197	30.9 (30.5–32.0)

**Figure 7.**
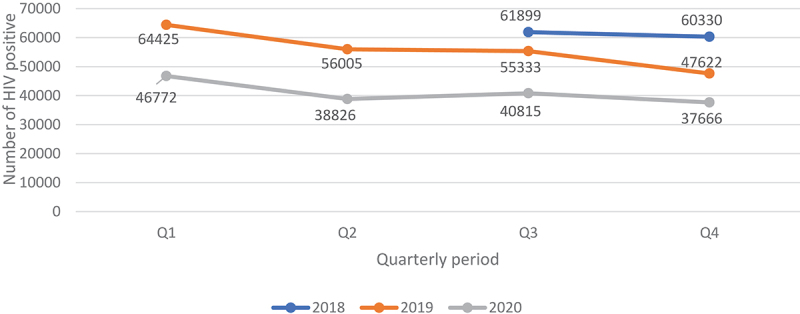
Quarterly data for people who tested HIV positive from July 2018 to December 2020.

Overall, annual recorded number of new PLHIV fell by 26.5% (95% CI 26.37–26.73) in 2020 compared to 2019. The proportionate drop was similar 26.2% when we compared Q2-Q4 of 2020 with similar period in 2019 (Figure 7). The proportionate change in those who tested positive in each of the quarters Q2-Q4 of 2020 relative to Q1 as a base period without COVID-19 was 17.0% drop in Q2, 12.7% drop in Q3 and 19.5% drop in Q4. However, the trend among those who tested HIV positive in 2020 was similar to the trend in 2019. Figures 4–6 show HIV testing numbers and positivity rate trends overall, by age and sex. Overall, annual HIV positivity rate was higher in 2020, 6.44% (95% CI 6.41–6.47) compared to 4.94% (95% CI 4.92–4.96) in 2019 and 6.62% (95% CI 6.58–6.66) in Q2-Q4 of 2020 versus 5.53% (95% CI 5.50–5.56) Q2-Q4 of 2019. Trends in positivity rate were low in Q3 and Q4 of 2018 around 3.8% where testing numbers were high. As testing numbers began to drop, HIV positivity rate began to rise reaching 4.5% in Q2 of 2019, then 6.5% in Q4 2019 and reaching a high of 6.8% in Q3 of 2020. By age and sex, positivity rate trends followed a similar trend as the overall. Annual positivity rate for adult males was 6.80% (6.75–6.85) in 2020 versus 5.56% (95% CI 5.52–5.60) in 2019 and 6.91% (95% CI 6.85–6.97) in Q2-Q4 of 2020 versus 6.02% (95% CI 5.97–6.07) Q2-Q4 of 2019, while for women it was 6.80% (95% CI 6.76–6.84) in 2020 versus 5.43% (95% CI 5.40–5.46) 2019 overall, and 6.99% (95% CI 6.94–7.04) 2020 versus 6.08% (95% CI 6.04–6.12) 2019 for Q2-Q4 period.

**Figure 8.**
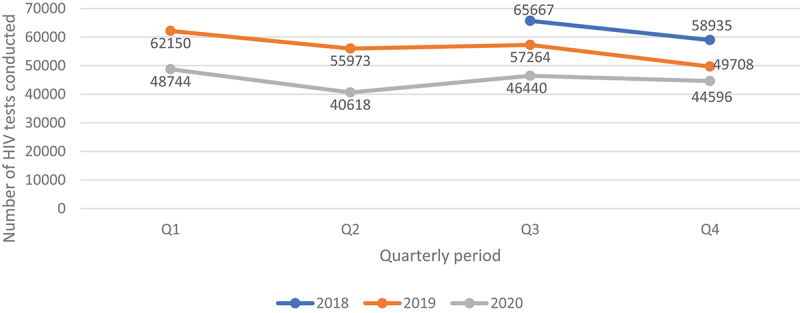
Quarterly ART initiation data for PLHIV who initiated ART between July 2018 and December 2020.

Annual drop in ART initiation was 19.9% (955 CI 19.7–20.0), (180 398 ART initiations) in 2020 compared to 225 095 ART initiations in 2019. The percentage drop remained similar (19.20%) when we compared Q2-Q4 of 2020 with similar period in 2019 (Figure 8). In 2019, the number of people who initiated ART 225 095 exceeded the number of people who tested HIV positive 223 385 in a calendar year (note that ART initiation and HIV positivity data are independent) by 1710, meaning 0.77% more PLHIV initiating ART than those tested positive, while in 2020 the number of people initiating ART, 180 398, exceeded the number who tested positive 164 079 by 16,319, meaning 10.0% more PLHIV were initiated on ART than those who tested positive.

**Figure 9.**
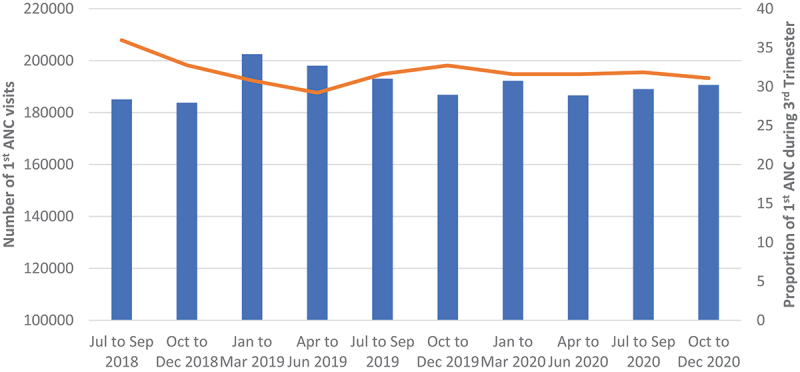
Showing quarterly first ANC visit for pregnant women.

**Figure 10.**
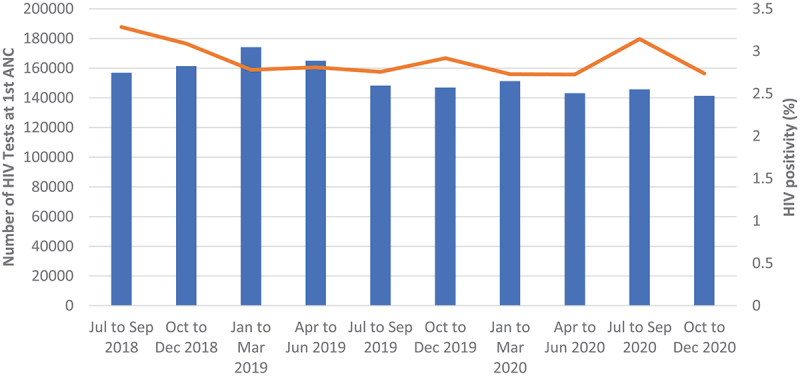
Showing quarterly HIV positivity at ANC.

The number of ANC visits remained relatively the same throughout the period of analysis (over 180 000 ANC visits), while the number of those who were tested for HIV slightly declined from about 160 000 in Q4 of 2018 to slightly above 140 000 tests in Q4 of 2020. HIV positivity rate at ANC was above 3.0% in the last two quarters of 2018, dropped to around 2.8% in Q1 of 2019, where it remained relatively unchanged for the rest of the analysis period (Figures 9 and 10).

**Figure 11.**
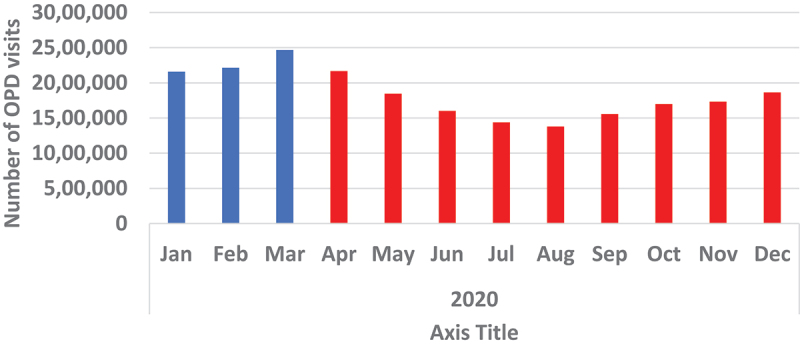
Showing monthly OPD visits for year 2020.

**Figure 12.**
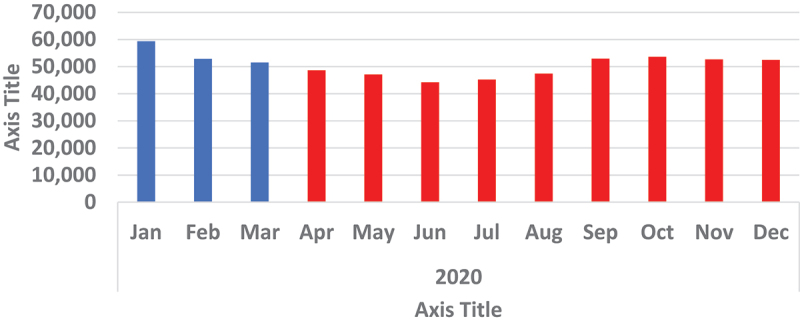
Showing monthly hospital admissions for year 2020.

Assessment of use of essential hospital services showed an increase in OPD visits from January of 2020, reaching its highest pick of the year at about 2.5 million OPD visits in March before starting a downward trend. The steady decline in OPD visits continued until August 2020. OPD visits slowly picked up again in September until December 2020, but the numbers had not returned to the levels seen earlier in the year. Hospital admissions took a different route. Starting from the highest point of about 59,000 admissions in January 2020, the numbers kept going down by month until June. In July, more people started to be admitted again and hospital admission stabilised at slightly above 50,000 admissions in a month from September to the end of 2020 (Figures 11 and 12).

## Discussion

HIV testing numbers dropped greatly during COVID-19 compared to pre-COVID-19 period in all three comparison periods. While HIV testing trend was on the decline throughout the analysis period, positivity yield increased as a result of MOH policy focusing on people at higher risk of HIV infection. However, quarterly trends on number of people who tested positive were similar between 2020 and 2019. There was a drop in ART initiation during COVID-19 compared to period without COVID-19. Nonetheless, in 2020, the number of PLHIV initiating ART exceeded the number who tested positive by a far greater margin than in 2019. At ANC, no major changes were observed in testing numbers and positivity yield remained relatively the same while OPD visits, and hospital admissions trends seemed to be influenced by COVID-19.

The decline in HIV testing numbers in Zambia started way back in 2018. This decline was attributed to the government and its implementing partners policies on HIV testing. The available evidence showed that many people who test positive already tested positive previously [23]. This evidence coupled with the need for efficient and cost-effective methods of testing to find remaining PLHIV had prompted governments and organisations to try other methods of finding the remaining PLHIV. The Zambian government developed an HIV risk screening tool to identify and test only those at high risk of contracting HIV. USAID was also supporting the MOH fight against HIV through USAID SAFE (Supporting an AIDS Free Eera) programme. Their programme which was implemented in three high prevalence provinces was successful in identifying many PLHIV [33]. However, it was also noted that risk screening tools do have challenges, and can miss PLHIV who don't know their status and decrease the absolute number of PLHIV diagnoses over-time. This programme, which focused on widespread testing to increase testing coverage at early stage of its inception, shifted to targeted testing through index client testing [34]. USAID used the screening tool first to screen males seeking VMMC in their Zambia DISCOVER-Health Project [35]. PEPFAR, which also supported the MOH in its quest to reduce testing, dropped community door-to–door testing because it had low positivity yield [20]. It then pushed for targeted testing for higher-risk individuals in their programmes by enhanced index testing and increased testing outside of health facilities [15]. In 2020, PEPFAR Zambia completely stopped supporting broad-based testing and prioritised targeted and index testing [15]. To boost men’s testing which was low compared to females, self-testing was expanded in 2020 after a successful pilot [15]. Other testing methods implemented by the government and its implementing partners over the last few years include campaigns or targeted testing for children and for adolescents and young people as these have some of the low testing rates in the country.

Since the implementation of these testing methods, the efforts were beginning to manifest through reduction in testing numbers while positivity yield was increasing [15,34,35] and a large number of testing kits were being saved [34]. In 2020, the USAID SAFE project reported an increase in positivity yield from 5.0% in March to 10.0% in September 2019 while testing numbers dropped by 67.0% when they started targeted testing. This is in comparison to a decline in positivity yield from 7.0% in October 2018 to 5.0% in March 2019 when their focus was to increase testing coverage through widespread testing [34]. Similarly, through another project, Zambia DISCOVER-Health Project [35], USAID reported an increase in positivity yield. Zambia achieved its annual HIV testing and ART initiation target for 2020 ahead of time. The target for ART initiation was set at 76,503, while testing was set at 5,460,136. This was despite facing the problem of COVID-19. The target figures for 2020, however, were set low to be in line with the changes in testing policy implemented prior. The new policies implemented dealt away with mass testing (over testing) which were not efficient and cost effective in favour of targeted testing which had been successful in its implementation.

Despite a drop in ART initiations during COVID-19 period compared to same period pre-COVID-19, more people were initiated on ART than those who tested positive during COVID-19 period. Zambia implemented the WHO universal treatment recommendations [22] allowing all PLHIV to be initiated on ART regardless of CD4 cell count in 2017 [36]. In theory, with time, the ratio of people who test positive to the people being initiated on ART should be around 1 or the ± difference should be smaller with years, but it was bigger in 2020 compared to 2019. COVID-19 could have played a role in the increased numbers being initiated on ART as PLHIV who already knew their status but were not on ART or had defaulted treatment may have wanted to boost their immune system by being on ART to protect against COVID-19.

ANC did not experience any disruption both in terms of number of testing and positivity yield. On the other hand, the use of essential hospital services, OPD and hospital admissions were affected. The drop in OPD numbers in April 2020 could be a direct result of first cases being diagnosed and announced in March. The fear of getting infected could have led many people to avoid places that were perceived as COVID-19 high-risk areas, therefore preferring to manage their mild illnesses at home. However, when the number of COVID-19 cases started to pick up during the first wave, hospital attendance and admissions started to go up as well. This increase could potentially be a direct result of many people being infected with COVID-19, thereby seeking hospital care and those in serious condition being hospitalised.

The nature of spread of COVID-19 which instilled fear in people and all the disruptions it brought through repurposing of resources including health personnel, closure of health facilities or services, governments imposition of lockdown or limitation on public transportation use restricting people’s movements meant that it was going to affect HIV testing and other health care services in a similar way Ebola did [24–30]. The Zambian government, however, did not respond with strict lockdown, neither were there any closures of health facilities or services leaving only people’s fears to contract the virus and restricted movement across district boundaries as the main direct links through which COVID-19 could affect HIV testing numbers. The government, however, encouraged its citizens to wear personal protective equipment (PPE) such as face masks when in public places, to observe social distancing and to practice hygiene. These interventions may have mitigated people’s fears in public spaces.

While we observed an increase in positivity yield due to targeted testing, we are not sure what proportion of those new positives were repeat testers who already knew their positive status. It would have been better if we had such information. Therefore, as targeted testing is being intensified, there is need to find ways of identifying especially those not aware of their status. It would also have been better to have data on self-testing, CD4 data for those initiating ART and data on specific entry point where people access HIV services as it would have given a more detailed understanding of the impact and which services were used most during COVID-19. In addition, the fact that data on testing and ART initiation were independent is another disadvantage as it was not possible to calculate what proportion of new positives were actually new or were returning to be initiated on ART because of test and treat or were defaulters who were returning to be reinitiated on ART. However, the availability of several years of data on testing, ART, and use of essential hospital services at national level, covering both periods before and during COVID-19 are the main strengths of this study.

## Conclusion

It is difficult to attribute the decline in testing numbers in Zambia to COVID-19 considering that the declining trend started way back in Q1 of 2019 as a result of MOH’s deliberate policies to reduce testing. COVID-19 may have contributed to low testing numbers during COVID-19 period, but its impact is very minimal given that the set targets on testing were met. The new policies on testing introduced before COVID-19 cases were reported in the country helped in reducing the number of people visiting testing sites in health facilities, effectively reducing overcrowding and promoting social distancing in testing facilities.

While COVID-19 had without a doubt a negative impact on health service delivery, Zambia HIV service delivery withstood its effects. HIV policies that were implemented before COVID-19 especially on testing helped the country to adopt COVID-19 control measures with ease and continued to provide HIV testing services without much disruption. The resilience of these policies is a positive for Zambia as it will continue to provide those services to people while keeping track on ending HIV as public health problem. We believe that this successful story in Zambia could be an example to follow for other countries who have struggled with providing HIV services to their population during the COVID-19 pandemic. The measures used in Zambia could help minimise the impact on HIV service delivery of future epidemics. It remains imperative for Zambia to continue to strengthen HIV testing and treatment efforts and leverage lessons learned during the COVID-19 pandemic.

## Paper context

The COVID-19 pandemic disrupted delivery of many services in many countries across the world. One area that was greatly affected is health service delivery especially HIV testing and care services in high-burdened countries. This article presents how Zambia’s HIV policies on testing that had been adopted and implemented before COVID-19 helped the country withstand the COVID-19 disruptive effects on HIV testing services and care.
